# Image-Based Grouping during Binocular Rivalry Is Dictated by Eye-Of-Origin

**DOI:** 10.1371/journal.pone.0095327

**Published:** 2014-07-02

**Authors:** Sjoerd M. Stuit, Chris L. E. Paffen, Maarten J. van der Smagt, Frans A. J. Verstraten

**Affiliations:** 1 Universiteit Utrecht, Neuroscience & Cognition Utrecht, Helmholtz Institute, Division of Experimental Psychology, Utrecht, The Netherlands; 2 University of Sydney, School of Psychology, Sydney, Australia; Harvard Medical School, United States of America

## Abstract

Prolonged viewing of dichoptically presented images with different content results in perceptual alternations known as binocular rivalry. This phenomenon is thought to be the result of competition at a local level, where local rivalry zones interact to give rise to a single, global dominant percept. Certain perceived combinations that result from this local competition are known to last longer than others, which is referred to as grouping during binocular rivalry. In recent years, the phenomenon has been suggested to be the result of competition at both eye- and image-based processing levels, although the exact contribution from each level remains elusive. Here we use a paradigm designed specifically to quantify the contribution of eye- and image-based processing to grouping during rivalry. In this paradigm we used sine-wave gratings as well as upright and inverted faces, with and without binocular disparity-based occlusion. These stimuli and conditions were used because they are known to result in processing at different stages throughout the visual processing hierarchy. Specifically, more complex images were included in order to maximize the potential contribution of image-based grouping. In spite of this, our results show that increasing image complexity did not lead to an increase in the contribution of image-based processing to grouping during rivalry. In fact, the results show that grouping was primarily affected by the eye-of-origin of the image parts, irrespective of stimulus type. We suggest that image content affects grouping during binocular rivalry at low-level processing stages, where it is intertwined with eye-of-origin information.

## Introduction

During binocular rivalry, dissimilar images presented dichoptically compete for perceptual awareness. One of the primary debates in rivalry research concerns the level of processing at which this competition originates. Evidence in favour of both an early, ‘eye-based’ and a later, ‘pattern-based’ level of processing has been presented over the years [1]–[4]. In recent years consensus seems to have been reached suggesting that rivalry competition occurs at multiple stages along the stream of visual information processing [4]–[9]. In spite of this consensus, the degree to which different processing levels contribute to rivalry remains elusive.

Several studies have shown that rivalry is the result of competition at neighbouring rivalry zones, whose competition is not independent. For example, adjacent rivalry zones tend to produce the same dominant percept when the rival targets share similar features such as motion, orientation or colour [10]–[13]. In other words, different regions of images engaged in rivalry can group together during dominance, resulting in a relatively stable, long-lasting dominance period. The term grouping is used here to refer to the simultaneous dominance of two particular images, or image parts, presented at different spatial locations. Neighbouring rivalry zones can group together based on the content of the presented images. This results in a percept of a coherent image, whose parts are presented to different eyes. Such an effect was first reported by Diaz-Caneja in 1928 [14], who presented the two halves of two coherent images (one image consisting of concentric lines and one consisting of straight lines) dichoptically, with matching halves presented to different eyes. Apart from perceiving the two different halves of the images (indicating that the input to a single eye produced the dominant percept), matching halves (i.e. concentric circles versus straight lines only) were also perceived. In other words, the two halves were grouped together in dominance to reconstruct a coherent image (also see [11]). Consequently, grouping during rivalry is usually associated with grouping based on image content, thereby reflecting pattern-based, higher-level competition [11 15–16]. However, this interpretation potentially obscures a different form of grouping, which is eye-based (e.g. perceiving the different halves of the images in Diaz-Caneja’s case). In a previous study, we set out to quantify and compare this grouping based on eye-of-origin to grouping based on image content [17].

In our previous study [17] we used a pair of rivalling horizontal and vertical gratings. The gratings with identical orientations could be presented to the same eye or to different eyes allowing us to estimate dominance durations based on both image-content and eye-of-origin. Interestingly, grouping of images presented to the same eye appeared to be much more potent than grouping based on image-content (i.e. by orientation). The effect of a shared eye-of-origin on dominance durations was also very prominent in a second experiment using diagonally oriented gratings. In contrast, grouping based on image-content was only present for cardinally oriented gratings. Together, these results suggest that dominance duration is primarily affected by the eye-of-origin of the presented images, but that there is also room for image-based cues to contribute to grouping. Since this study [17] only used oriented gratings, it is possible that the image-based cues where not potent enough to contribute to grouping, leading to an underestimation of the contribution of this cue. This possible pitfall is our main concern in the current study. Does the contribution of image-based grouping during rivalry increase when the image-content is biased towards images that are known to be processed relatively late in the visual processing stream?

In the first experiment of the current study we address the question whether image-based grouping increases for images that are known to be processed relatively late in the visual processing hierarchy compared to those that are processed relatively early. The feature-preferences of neurons become more complex throughout the visual processing hierarchy. While the early visual cortex shows tuning to simple orientation, later areas respond to more specific stimuli like objects [18], places, or faces [19]. For example, the Fusiform Face Area (FFA) has been shown to respond preferentially to faces [19]. Moreover, this latter area is thought to respond to faces as a whole [20], rather than just a collection of the parts of the face. Also, it responds preferentially to upright faces, in comparison to inverted faces [21]. These characteristics make faces an ideal stimulus to enhance grouping based on image content. If simultaneous dominance (i.e. grouping) is affected by higher-level face processing, we can expect a bias towards perceiving image-based grouped faces since they are processed as a whole. Alternatively, grouping during rivalry may be unaffected by such relatively late processes.

To compare image-based grouping for higher-level stimuli to stimuli processed at the lower end of the visual processing stream, we also used oriented gratings. Grouping for these kind of stimuli is known to be primarily eye-based. We also used inverted faces, as they do not activate higher-level visual processing areas as much as upright faces [21].

## Methods Experiment 1

### Participants

A total of 7 participants, including one of the authors (SS) participated in the experiment. This study involves healthy human participants, and does not utilize any invasive techniques, substance administration or psychological manipulations. Therefore, compliant with Dutch law, this study only required, and received approval from our internal faculty board (Faculty’s Advisory Committee under the Medical Research (Human Subjects) Act (WMO Advisory Committee) at Utrecht University. Furthermore, this research was conducted according to the principles expressed in the Declaration of Helsinki. All participants in the experiment had provided written informed consent. In doing so, they had indicated to have read and to have agreed with both the rules regarding participation and proper (laboratory) behavior, and the researchers’ commitments and privacy policy. They were also informed that they could stop participating in the experiment whenever they wanted to do so and that all data would be analyzed anonymously. All participants had normal or corrected to normal vision and passed a test for stereo-vision (TNO test for stereoscopic vision). With the exception of SS, the participants were naïve as to the purpose of the experiment.

### Apparatus

Stimuli were created on an Apple - Mac Pro computer running Matlab 7.4 with the Psychophysics Toolbox extensions [22]–[23]. The stimuli were presented on a linearized LaCie III 22″ at 75 Hz. Participants viewed the stimuli through a mirror stereoscope. The length of the optical path was 57 cm.

### Stimuli

**Figure 1:**
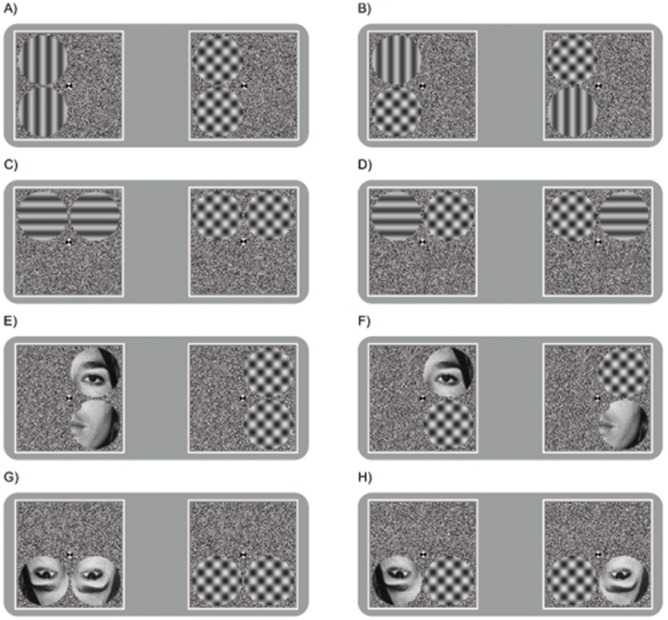
Schematic examples of the competing stimuli used in Experiment 1. Identical or matching images were presented in four different conditions: 1) Matching images presented to the same eye and the same hemifield (A/E). 2) Matching images presented to the same eye and in different hemifield (C/G). 3) Matching images presented to different eyes and in the same hemifield (B/F). 4) Matching images presented to different eyes and in different hemifields (D/H).

Experiment 1 consisted of 2 parts, which were run separately. For part 1 we used grating stimuli (Experiment 1: Gratings; Figure 1A–D), for part 2 we used face stimuli (Experiment 1: Faces; Figure 1E–H). Specifically, the rivalling images consisted of horizontal and vertical sine-wave gratings or parts of faces presented to one eye, paired with plaids presented to the other eye. The gratings were presented at a Michelson contrast of 49.7% with a space-average luminance of 25 cd/m^2^. The gratings had a spatial frequency of 4.1 cpd. For the parts of the neutral faces we used a male and a female taken from the Ekman and Friesen [24] face stimuli set. Plaids were presented at 74.4% Michelson contrast and had the same spatial frequency as the gratings. All interocular pairs were shown in circular apertures with a radius of 1.9° of visual angle whose edges were softened by a cosine ramp of 0.2° of visual angle, and were presented on a random pixel noise background of 98% (Michelson) contrast (25 cd/m^2^) that was identical in both eyes. The half-images were presented within square white frames. We used four basic stimulus arrangements in our experiments (Figure 1): 1) matching images in the same hemifield – in the same eye, 2) matching images in different hemifields – in the same eye, 3) matching images in the same hemifield – in different eyes, and 4) matching images in different hemifields – in different eyes. The distance from the fixation point to the centre of the target was 2.1° of visual angle and identical for all targets in all conditions.

### Procedure

Participants performed the experiment in a darkened room with their heads supported by a chin rest. Before the onset of each trial, participants were presented with two identical pixel noise half-images surrounded by white frames. At the centre of each half-image was a fixation point. When ready, a participant initiated a trial by pressing the space-bar key. Next, two gratings (either both horizontal or both vertical) or two parts of a face (upright or inverted) were presented in one of four possible spatial arrangements with two plaids in corresponding locations of the other eye. Participants performed a 3AFC perceptual tracking task where their task was to continuously indicate via a key press whether they perceived one plaid (left arrow key), two plaids (right arrow key) or no plaids (no press). Each trial lasted 45 s. After each trial, the rivalling targets were removed from the screen. Participants were instructed to fixate on the fixation point throughout the experiment. The experiment typically lasted about 140 minutes and was completed in 8 blocks.

### Analyses

For our main analyses we ran repeated measures ANOVAs on two data sets for both parts (grating- and face-stimuli) of the experiment: individual epoch durations and overall fraction of time a particular percept was dominant during a trial. For epoch duration we used the median of epochs per condition. This parameter is very informative about percept stability. For the fractions of overall dominance we used the mean fraction per condition. Fractions are very informative about biases towards certain percepts. Note that, for 3AFC-paradigms, results based on epoch durations and overall fractions may differ. For example, a particular percept may last only briefly (resulting in a short epoch duration) but occur very often (resulting in a large overall fraction).

Our planned comparisons focus on the differences between the results when using grating-stimuli and when using face-stimuli. Namely, we set out to test the differences (using paired-samples t-tests) between the average median epoch durations and the average fractions overall dominance for 1) grouped gratings and grouped faces, 2) grouped plaids in Experiment 1 and grouped plaids in Experiment 2, and 3) mixed percepts of a grating and a plaid and mixed percepts of a face-part and a plaid. These comparisons were done for both within- and between-eye dominance. We set our α to 0.0085 based on the Šidák [25] correction to adjust for multiple [6] comparisons per data type (epoch durations and fractions overall dominance).

## Results Experiment 1

For our analyses we extracted the median dominance epoch duration and fractions of total dominance duration for each of the three possible perceptual outcomes. When using grating-stimuli, these were responses indicating grouped gratings, grouped plaids and mixed percepts of one grating and one plaid. When using face-stimuli, these were percepts of grouped face-parts, grouped plaids and mixed percepts of one face-part and one plaid. A 2 (image orientation)×2 (within- vs. between eye dominance)×2 (within- vs. between hemifield dominance) repeated measures ANOVA was performed for each perceptual outcome. Tables 1 and 2 show a summary of the test statistics for grating- and face-stimuli respectively.

**Table 1:** A summary of the analyses of Experiment 1 when using grating-stimuli.

Percept	Gratings (0 response)						Plaids (2 plaids response)						Mixed (1 plaid response)					
Data type	Median			Fraction			Median			Fraction			Median			Fraction		
Statistic	F	p	_p_η^2^	F	p	_p_η^2^	F	p	_p_η^2^	F	p	_p_η^2^	F	p	_p_η^2^	F	p	_p_η^2^
**Image orientation (IO)**	0.04	.84	.01	5.85	>.05	.50	3.89	.10	.39	11.32	.02	.65	0.05	.83	<.01	7.45	.03	.55
**Eye**	4.87	.07	.45	**18.36**	**<.01**	**.75**	**10.30**	**.02**	**.63**	**27.63**	**<.01**	**.82**	**10.87**	**.02**	**.64**	**25.37**	**<.01**	**.81**
**Hemifield**	0.11	.75	.02	0.51	.50	.08	<0.01	.97	.00	8.69	.03	.59	4.24	.09	.41	3.93	.10	.40
**IO by eye**	3.92	.10	.40	2.34	.18	.28	1.79	.23	.23	0.07	.80	.01	0.54	.49	.08	0.91	.38	.12
**IO by hemifield**	0.85	.39	.12	2.11	.20	.26	<0.01	.96	<.01	3.30	.12	.36	0.39	.56	.06	.33	.58	.05
**Eye by hemifield**	4.23	.09	.41	**49.34**	**<.01**	**.89**	4.51	.08	.43	**15.76**	**<.01**	**.72**	**12.08**	**.01**	**.70**	**38.52**	**<.01**	**.87**
**IO by eye by hemifield**	0.62	.64	.09	3.21	.12	.12	0.84	.39	.12	5.35	.06	.47	0.52	.50	.08	0.13	.73	.02

F- & p-values as well as the partial eta squared (Statistic) for both median durations and fractions of overall dominance (Data type) are reported for the three possible perceptual outcomes (Percept). The different comparisons are noted in the left-most column. Significant effects are printed in bold. Note that image orientation for plaids refers to the orientation of the suppressed images.

**Table 2:** A summary of the analyses of Experiment 1 when using face-stimuli.

Percept	Face (0 response)						Plaids (2 plaids response)						Mixed (1 plaid response)					
Data type	Median			Fraction			Median			Fraction			Median			Fraction		
Statistic	F	p	_p_η^2^	F	p	_p_η^2^	F	p	_p_η^2^	F	p	_p_η^2^	F	p	_p_η^2^	F	p	_p_η^2^
**Image orientation (IO)**	1.51	.27	.20	5.18	.06	.46	2.42	.17	.29	3.29	.12	.35	**12.71**	**.01**	**.68**	1.74	.24	.23
**Eye**	**14.75**	**<.01**	**.71**	**35.87**	**<.01**	**.86**	5.32	.06	.47	**14.39**	**<.01**	**.71**	**31.43**	**<.01**	**.84**	**30.33**	**<.01**	**.84**
**Hemifield**	.83	.40	.12	**11.77**	**.01**	**.66**	**13.95**	**.01**	**.70**	**15.38**	**<.01**	**.72**	**9.82**	**.02**	**.62**	**29.86**	**<.01**	**.83**
**IO by eye**	3.65	.11	.38	1.44	.28	.19	<.01	.98	<.01	0.87	.39	.13	1.57	.26	.21	2.62	.16	.30
**IO by hemifield**	0.06	.81	.01	**7.75**	**.03**	**.56**	1.32	.29	.18	0.76	.42	.11	5.68	.05	.49	7.90	.03	.57
**Eye by hemifield**	0.20	.67	.03	**28.58**	**<.01**	**.83**	3.89	.10	.39	**43.58**	**<.01**	**.88**	**17.58**	**<.01**	**.75**	**112.96**	**<.01**	**.95**
**IO by eye by hemifield**	0.01	.92	<.01	0.06	.81	.01	1.20	.32	.17	0.03	.88	<.01	2.19	.19	.27	0.17	.70	.03

F- & p-values as well as the partial eta squared (Statistic) for both median durations and fractions of overall dominance (Data type) are reported for the three possible perceptual outcomes (Percept). The different comparisons are noted in the left-most column. Significant effects are printed in bold. Note that the orientation of the faces did not affect their epoch durations or the overall dominance (IO).

### Epoch durations: image-orientation and eye-of-origin effects

**Figure 2:**
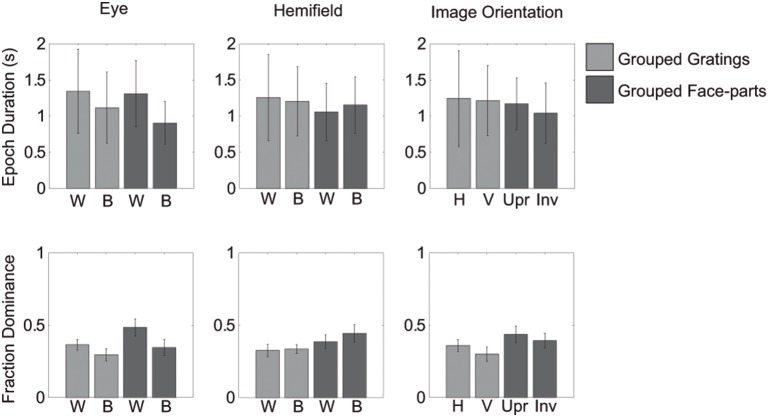
Main effects of Experiment 1. Overview of main effects of within- versus between eye dominance, within- versus between hemifields and image orientation for grouped gratings (light grey) and grouped face-parts (dark grey). Error bars represent the 95% confidence intervals. The 3 top panels depict the average median epoch duration in seconds. Each participant’s data was normalized using his or her overall median dominance duration. After averaging across participants the averages were multiplied by the overall median across all participants, which results in a duration in seconds. Note that this normalization used was only for graphical purposes. The other 3 panels depict the fraction of overall dominance. The different conditions are denoted on the abscissa as follows W: within- eye/hemifield, B: between- eye/hemifield, H: horizontal, V: vertical, Upr: upright, Inv: Inverted.

Importantly, in only one condition did we find an effect of image-orientation (IO, mixed percept in table 2): here the combination of a plaid and an inverted face-part lasted an average of 0.17 (standard error: 0.05) seconds longer than a plaid and an upright face-part. Note that in addition to the small size of the effect its direction is opposite to our hypothesis: Upright faces were assumed to results in longer dominance durations than inverted faces due to their relatively late processing locus. Except for this effect on mixed-percept durations when using face-stimuli, no main effect of image-orientation was apparent in either experiment (‘IO’: all perceptual outcomes in tables 1&2). This means that horizontal and vertical gratings did not differ in their epoch duration or fractions of overall dominance. Likewise, upright and inverted face-parts did not differ in their epoch duration or fractions of overall dominance either. Instead, grouping during dominance appears to be affected primarily by eye-of-origin of the dominant images (the same eye or different eyes; ‘Eye’, all perceptual outcomes in tables 1&2). Specifically, images that were presented to the same eye tended to be dominant together for longer periods and an overall larger proportion of time (see Figure 2).

### Epoch durations: hemifield effects

**Figure 3:**
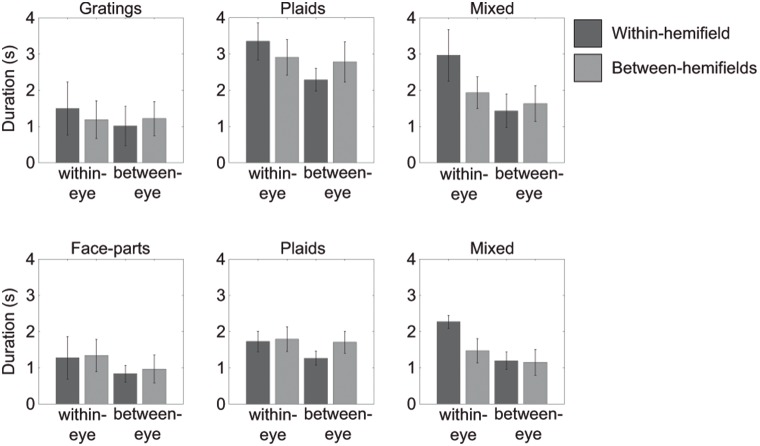
Median percept durations in Experiment 1. Within-hemifield dominance durations are presented in dark grey. Between-hemifield dominance durations are presented in light grey. Error bars represent the 95% confidence intervals. For illustration purposes only, durations were normalized. Each participant’s data was normalized by his or her overall median dominance duration. After averaging across participants the averages were multiplied by the overall median across all participants, which results in a duration in seconds. Note that between-eye dominance of gratings, plaids and face-parts reflects grouping based only on image-content. Within-eye dominance for mixed-percepts reflects grouping based only on eye-of-origin. Within-eye dominance for gratings, plaids and face-parts reflect a combination of eye-of-origin- and image-based grouping.

**Figure 4:**
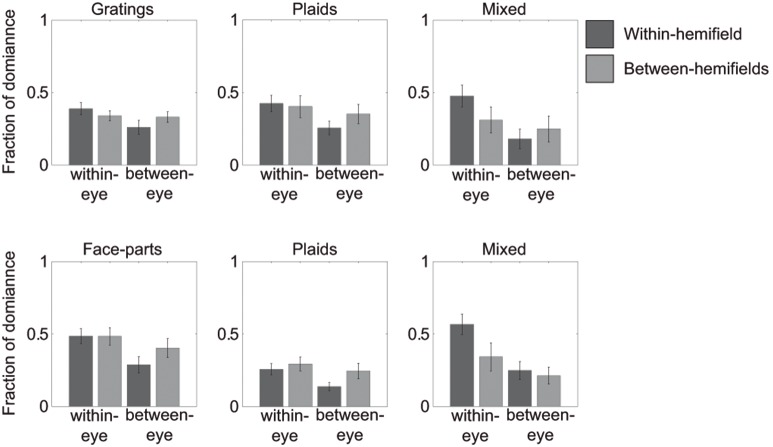
Fractions of overall dominance in Experiment 1. Within-hemifield fractions of overall dominance are presented in dark grey. Between-hemifield dominance fractions of overall are presented in light grey. Error bars represent the 95% confidence intervals. Note the overall pattern in the data is similar to the epoch duration data.

When using face-stimuli, we found main effects for the location in the visual field of the dominant images (i.e. within- versus between hemifields). For both dominant face parts and plaids, images in different hemifields resulted in longer simultaneous dominance than images in the same hemifield (Hemifield in table 2; also see Figure 2 & 3). However, for a mixed face-plaid percept, images were dominant together more within the same hemifield (Figure 3). For both grating- and face-stimuli, we mainly found interactions between the eye-of-origin of the dominant images and their placement across the visual field (Eye by hemifield interaction in table 1 and table 2; also see Figure 3). Specifically, when using gratings, images presented to the same eye appeared to be dominant together longer when presented in the same hemifield, compared to different hemifields. However, when the images were presented to different eyes, they were dominant together longer when in different hemifields, compared to the same hemifield (Figure 3). These finding replicate our previous work on grouping [17]. When using face-stimuli, interactions between the arrangement across the visual field and eye-of-origin of the dominant images were also apparent (Figure 3 & 4). However, this relationship appears to be affected by the presence of a main effect of how the items are arranged across the visual field (i.e. the hemifield condition). We suggest the main hemifield effect and the hemifield by eye interaction have opposite effects on *within*-eye grouping durations for face-parts and plaids. This results in the effect of hemifield appearing smaller for within-eye dominance. For mixed face-plaid percepts, the main hemifield effect is opposite to the main hemifield effect for grouped faces and grouped plaids: for mixed percepts, within-hemifield grouping is more prominent instead of between-hemifield grouping. Now, the main hemifield effect and the hemifield by eye interaction have opposite effects on *between*-eye grouping. As a result, the effect of hemifield appears smaller for between-eye dominance (Figure 3 & 4).

### Epoch durations: image-based effects

The main aim of Experiments 1 was to test whether more image-based grouping occurs when using images that are processed higher up the visual processing hierarchy (i.e. that require neural machinery beyond early visual cortex). The lack of differences in grouping upright versus inverted face-parts suggests that the proposed higher-level processing of faces does not contribute to a large extent to grouping during rivalry. However, since inverted faces can still be considered ‘more complex’ and therefore might still have been processed at relatively late processing stages, albeit to a lesser extent than upright faces [21], we also compared faces directly to gratings. To this end, we focused on the difference between dominance durations for grouped gratings and grouped parts of faces (irrespective of grating or face orientation). Epoch durations did not differ between the grouped gratings and faces during within-eye dominance (*t(1,6) = 0.5405, p = .608*) nor during between-eye dominance (*t(1,6) = 2.1362, p = .077*; see also Figure 3). Note that the comparison of between eye-dominance of the two percepts is a direct comparison of image-based grouping, since image-complexity is the only possible source of a difference in grouping of these images.

### Epoch durations: plaid- and mixed-percepts

The results of Experiment 1 suggest a limited effect of the type of dominant images used on grouping durations. However, we did find that epoch durations of dominant plaids were shorter when face-parts, in comparison to gratings, were suppressed (*within-eye dominance: t(1,6) = 4.2748, p = .005, between-eye dominance: t(1,6) = 5.0349, p = .002*; see Figure 3). This result suggests that plaids group together better during dominance when gratings are suppressed. Alternatively, this could also suggest that face-parts are more potent in breaking suppression than gratings. In light of previous results showing shorter suppression durations for emotional faces compared to neutral faces [26]–[28], we suggest the latter option to be more plausible. No differences were found for mixed percept epoch durations depending on the stimulus type (gratings compared to faces; *within-eye dominance: t(1,6) = 2.5884, p = .041, between-eye dominance: t(1,6) = 2.6613, p = .038*; see Figure 3).

### Fractions of overall dominance

Finally, we compared the fraction of overall dominance for the different percepts when using gratings compared to using faces. Results show that grouped face-parts are perceived for a larger portion of time than grouped gratings (*within-eye dominance: t(6) = −5.7579, p = .001;* see Figure 4). Importantly, this difference was only present for within-eye dominance (*between-eye dominance: t(6) = −2.6976, p = .036;* Figure 4). This result shows that within-eye grouping effects are stronger for faces compared to gratings. Similar to the results for epoch durations, grouped plaid percepts occurred for a larger portion of time when gratings were simultaneously suppressed as compared to when faces were suppressed (*within-eye dominance: t(6) = 5.9340, p = .001, between-eye dominance: t(6) = 4.1099, p = .006*; Figure 4). No differences were found between the overall occurrences of mixed percepts between the two experiments (*within-eye dominance: t(6) = −0.5809, p = .582, between-eye dominance: t(6) = −1.7484, p = .131*; see Figure 4).

### Conclusions Experiment 1

Taken together, these results suggest that grouping during binocular rivalry dominance is primarily affected by the source, that is the eye-of-origin, and the relative positions in the visual field of the grouped images. Grouping is not different for higher-level image modulations such as face-inversion. We did, however, find a difference between the overall durations for perceiving grouped faces and perceiving grouped gratings. Importantly, this effect was only present for within-eye dominance, showing that different image content can increase the overall duration of eye-based dominance.

## Experiment 2

In Experiment 1 we did not observe an increase in image-based grouping for upright faces when comparing them to inverted faces. Nor was such an increase apparent when individual epoch durations between faces and gratings were compared. In contrast, a shared eye-of-origin resulted in more grouping during dominance for all percepts. Note that this finding is in line with the results from our previous work [17]. We also found variations in dominance durations based on differences in the arrangement across the visual field, and again these effects concur with our previous findings.

In the next experiment we will explore a different approach to test for an increase in the contribution of image-based grouping. We will use depth cues to manipulate amodal completion; when an object is occluded by another object, the occluded object’s shape can be amodally completed without sensory input [29]. Relative depth can have a profound effect on amodal completion by manipulating border ownership [30]. The common border between the occluder and the occluded object is referred to as *intrinsic* to the occluder and *extrinsic* to the occluded object. Moreover, borders considered intrinsic to an object are argued to hinder grouping, while extrinsic borders facilitate grouping [30]. Here we use the relative depth of the surrounding background and the rivalling items to create conditions where the items appear occluded or non-occluded, by shifting the background in depth toward or away from the observer. We expected that manipulating amodal completion with border ownership would affect image-based grouping specifically since eye-based grouping is thought to be unaffected by image-content [17]. Moreover, if amodal completion facilitates image-based grouping, the effect may be largest for upright faces. That is, as compared to gratings and inverted faces, since this stimulus is considered to be the most complex, and requires high-level image-based processing.

## Methods Experiment 2

### Participants

10 participants, including 3 participants from the previous experiment, participated in Experiment 2. This study involves healthy human participants, and does not utilize any invasive techniques, substance administration or psychological manipulations. Therefore, compliant with Dutch law, this study only required, and received approval from our internal faculty board (Faculty’s Advisory Committee under the Medical Research (Human Subjects) Act (WMO Advisory Committee) at Utrecht University. Furthermore, this research was conducted according to the principles expressed in the Declaration of Helsinki. All participants in the experiment had provided written informed consent. In doing so, they had indicated to have read and to have agreed with both the rules regarding participation and proper (laboratory) behavior, and the researchers’ commitments and privacy policy. They are also informed that they can stop participating in the experiment whenever they want to do so and that all data would be analyzed anonymously. All had normal or corrected to normal vision and all but one were naïve as to the purpose of the study. All participants passed a test for stereo-vision and reported perceiving the correct depth ordering in the stimuli used for this experiment.

### Apparatus

The materials and software used were identical to Experiment 1.

### Stimuli & Procedure

**Figure 5:**
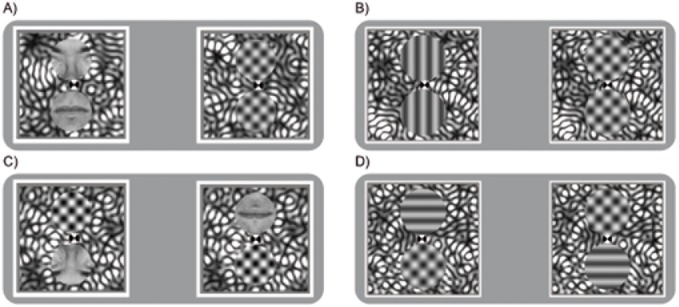
Schematic representation of the stimuli used in Experiment 2. Matching images were presented along the vertical meridian and could be presented to the same eye (A&B) or to different eyes (C&D). For the matching images we used upright (A) and inverted (C) faces, as well as vertical (B) and horizontal gratings (D). The surround was presented with either crossed or uncrossed disparity, resulting in the images being perceived as either being occluded by the surround or not.

The stimuli and procedure used in Experiment 2 were identical to those used in the first experiment, with the following exceptions: All stimuli were presented along the vertical meridian (see Figure 5). The hemifield condition was removed to focus solely on eye- and image-based contributions. For the noise-background we used band-pass filtered pixel noise. The background was presented with a crossed and uncrossed disparity of 10 min/arc to achieve the percept of occluded or non-occluded rivalling images. To keep the task focused on the plaids, we now included a ‘zero-plaids-visible’ response button. This response button was added to remove a potential bias towards percepts that contained plaids. Participants thus used 3 keys in the perceptual tracking task instead of 2. Furthermore, trials lasted 60 s instead of 45 s, making the total duration of Experiment 2 identical to Experiment 1.

## Results Experiment 2

**Figure 6:**
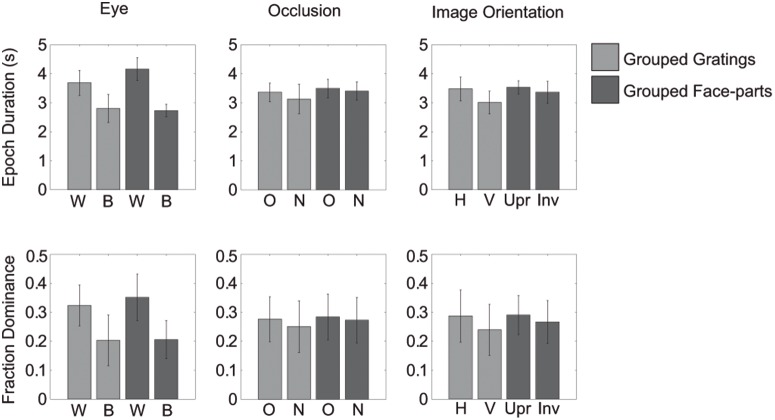
Main effects of Experiment 2. Overview of main effects for within- versus between eye dominance, occluded versus not occluded and image orientation for grouped gratings (light grey) and grouped face-parts (dark grey). Error bars represent 95% confidence intervals. The top three panels depict the average median epoch duration for grouped gratings (light grey) and grouped face-parts (dark grey). Each participant’s data was normalized using his or her overall median dominance duration. After averaging across participants the averages were multiplied by the overall median across all participants, which resulted in a duration in seconds. Note that the data normalization was for graphical purposes only since we used a within-subjects statistical design. The bottom three panels depict the fractions of overall dominance for grouped gratings and grouped face-parts. The different conditions are denoted on the abscissa as follows: W: within-eye, B: between-eyes, O: occluded, N: not occluded, H: horizontal, V: vertical, Upr: upright, Inv: Inverted. Note that between-eye grouping (left panels, conditions B) reflects grouping based on image-content only.

**Figure 7:**
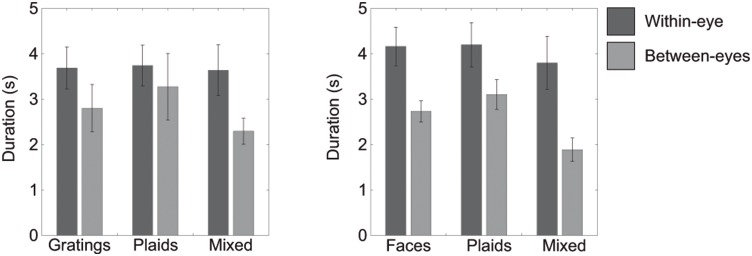
Median percept durations for Experiment 2. Within-eye dominance durations are presented in dark grey. Between-eye dominance durations are presented in light grey. Error bars represent the 95% confidence intervals. For graphical purposes the data was normalized by each participant’s overall median duration before being averaged across subjects and then multiplied by the overall median across all participants. Note the strong dependency on the eye-of-origin of the dominant images.

**Figure 8:**
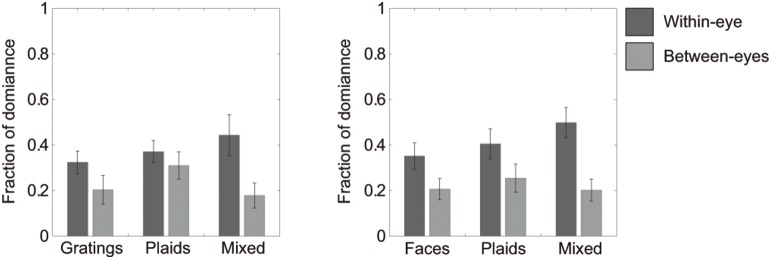
Fractions of overall dominance for Experiment 2. Within-eye dominance is presented in dark grey. Between-eye dominance is presented in light grey. Error bars represent the 95% confidence intervals. Note that the dependency on the source (i.e. the eye-of-origin) of the dominant images is similar to the epoch duration data.

For our analyses we extracted the median dominance epoch durations and fractions of total dominance duration for each of the possible perceptual outcomes. A 2 (occluded versus not occluded)×2 (image orientation)×2 (within- versus between eye dominance) repeated measures ANOVA was performed for each percept. That is, grouped face-parts or gratings, grouped plaids and mixed percepts of one face-part or grating and one plaid. The statistical results are summarized in table 3 (for grating-stimuli) and table 4 (for face-stimuli). We found neither a main effect of occlusion, nor any interactions between occlusion and any other condition on grouping during rivalry dominance (Occlusion, Occlusion by IO, Occlusion by eye & Occlusion by IO by eye in tables 3 and 4; Figure 6 upper and lower middle panels). This result suggests that amodal completion does not affect grouping during rivalry dominance. However, within-eye dominance resulted in longer epoch durations and more overall dominance than between-eye dominance for all percepts (Eye in tables 3 and 4; figures 7 and 8). As was true for the first two experiments, these results show a bias towards perceiving images presented to the same eye. These images are grouped together longer at the level of individual epochs as well as have longer overall durations (see figures 7 and 8).

**Table 3:** A summary of the analyses results of Experiment 2 when using gratings.

Percept	Gratings (0 response)						Plaids (2 plaids response)						Mixed (1 plaid response)					
Data type	Median			Fraction			Median			Fraction			Median			Fraction		
Statistic	F	p	_p_η^2^	F	p	_p_η^2^	F	p	_p_η^2^	F	p	_p_η^2^	F	p	_p_η^2^	F	p	_p_η^2^
**Occlusion**	0.86	.38	.09	1.07	.33	.11	0.94	.36	.10	0.03	.87	<.01	0.04	.85	>.01	0.48	.51	.05
**Image Orientation (IO)**	4.30	.07	.32	2.35	.16	.21	.20	.66	.02	1.58	.24	.15	0.04	.85	>.01	<.01	.93	<.01
**Eye**	**7.52**	**.02**	**.46**	**42.72**	**<.01**	**.83**	**14.35**	**<.01**	**.62**	**30.98**	**<.01**	**.78**	**16.78**	**<.01**	**.65**	**39.40**	**<.01**	**.81**
**Occlusion by IO**	1.94	.20	.18	3.02	.12	.25	<.01	.95	<.01	.22	.65	.02	1.99	.19	.18	0.57	.47	.06
**Occlusion by eye**	0.04	.85	<.01	1.03	.34	.10	1.27	.29	.12	3.05	.12	.25	0.50	.50	.05	4.70	.06	.34
**IO by eye**	2.04	.19	.19	0.34	.57	.04	0.11	.75	.01	1.08	.33	.11	0.30	.60	.03	0.36	.56	.04
**Occlusion by IO by eye**	0.26	.62	.62	2.71	.13	.23	1.00	.34	.10	1.54	.25	.15	.40	.55	.04	<.01	.99	<.01

F- and p-values as well as the partial eta squared (Statistic) for both median durations and fractions of overall dominance (Data type) are reported for the three possible perceptual outcomes (Percept). The different comparisons are noted in the left-most column. Significant effects are printed in bold. Note that significant differences are only present between within- and between-eye dominance.

**Table 4:** A summary of the analyses of Experiment 2 when using faces.

Percept	Face (0 response)						Plaids (2 plaids response)						Mixed (1 plaid response)					
Data type	Median			Fraction			Median			Fraction			Median			Fraction		
Statistic	F	p	_p_η^2^	F	p	_p_η^2^	F	p	_p_η^2^	F	p	_p_η^2^	F	p	_p_η^2^	F	p	_p_η^2^
**Occlusion**	0.49	.50	.05	.24	.64	.03	0.02	.90	<.01	1.01	.34	.10	2.49	.15	.22	0.70	.42	.07
**Image Orientation (IO)**	0.72	.42	.07	**10.74**	**.01**	**.54**	0.65	.44	.07	0.38	.55	.04	0.85	.38	.09	1.30	.28	.13
**Eye**	**58.29**	**<.01**	**.87**	**106.30**	**<.01**	**.92**	**18.14**	**<.01**	**.67**	**40.91**	**<.01**	**.82**	**53.55**	**<.01**	**.87**	**167.02**	**<.01**	**.95**
**Occlusion by IO**	0.25	.63	.03	0.26	.62	.03	0.18	.68	.02	0.64	.45	.07	0.39	.55	.04	0.97	.35	.10
**Occlusion by eye**	<.01	.99	<.01	0.07	.80	<.01	0.02	.90	<.01	0.39	.55	.04	1.34	.28	.13	0.45	.52	.05
**IO by eye**	0.85	.38	.09	2.30	.60	.03	0.60	.46	.06	2.10	.18	.19	0.01	.91	<.01	2.60	.14	.22
**Occlusion by IO by eye**	0.48	.51	.05	0.04	.84	<.01	.31	.59	.03	.22	.65	.02	0.35	.57	.04	<.01	.93	<.01

F- and p-values as well as the partial eta squared (Statistic) for both median durations and fractions of overall dominance (Data type) are reported for the three possible perceptual outcomes (Percept). The different comparisons are noted in the left-most column. Significant effects are printed in bold. Note that the most common significant effects are between within- and between-eye dominance.

### Effects of image-orientation

In contrast to Experiment 1, in Experiment 2 we *do* find a significant difference between the fractions of dominance for upright versus inverted faces (IO in table 4). This difference reflects a small bias toward perceiving upright face-parts compared to inverted face-parts (Figure 6, lower right panel). This bias is apparent for 9 out of 10 participants, but the magnitude was limited to a difference between fractions of 0.024 (standard error of the difference: 0.007). At the level of individual epoch durations, however, the upright face-parts were not perceived for longer consecutive periods than the inverted face-parts.

### Epoch durations: image-based grouping effects

In post-hoc comparisons, we compared the medians of individual epoch durations between using grating- and face-stimuli (see Figure 7), using the same planned comparisons as for Experiments 1. We found no differences between epoch durations for grouped faces compared to gratings (*within-eye dominance: t(9) = 2.5401, p = .032; between-eye dominance: t(9) = −0.2882, p = .779*). The epoch durations for grouped plaids when using gratings did also not differ from the epoch durations for grouped plaids when using faces (*within-eye dominance: t(9) = −1.4667, p = .176; between-eye dominance: t(9) =  − 0.4399, p = .670*). Likewise, epoch durations for mixed percepts did not differ depending on the stimuli used (gratings compared to faces; *within-eye dominance: t(9) = −0.5766, p = .578; between-eye dominance: t(9) = 2.9835, p = .015*).

### Fractions dominance: image-based grouping effects

Next, we compared the fractions of overall duration for grouped faces to those of grouping oriented gratings (Figure 8). Contrary to the comparisons between the results for the first experiment, there was no difference between the fractions of overall durations for gratings and faces (*within-eye dominance: t(9) = 0.8761, p = .404; between-eye dominance: t(9) = 0.0802, p = .938*). These results suggest that there is no difference between the occurrence of grouping gratings and faces. Also in contrast to Experiment 1, we did not find any difference in the durations for grouping plaids (*within-eye dominance: t(9) = −1.0896, p = .304; between-eye dominance: t(9) = 1.7034, p = .123*). Apparently, the increased duration for dominant plaids that depended on the suppressed image is not consistent across experiments. Our final comparison showed no differences between mixed-percept durations when using faces compared to gratings (*within-eye dominance: t(9) = −2.2665, p = .049; between-eye dominance: t(9) = −1.3419, p = .213*). These results do not indicate any higher-level involvement in grouping during rivalry.

## General Discussion

In the current study we investigated whether complex images that require processing at relatively late stages of the visual hierarchy increase the potential of image-based grouping during binocular rivalry. Our previous results demonstrated that image-based grouping can essentially be reduced to zero, while eye-based grouping remains strong when grating stimuli are used [17]. Specifically, in that study, we found an increase in dominance durations based image-content for cardinally oriented gratings, but not for diagonally oriented gratings. However, the images used in that experiment always consisted of simple gratings, which are already well processed in early visual areas. To overcome this possible limitation, we now also used parts of faces, either upright or inverted. Moreover, the competing images were presented with and without disparity-based occlusion. Using images that are believed to rely on later processing stages (such as the IT complex; [19]) as well as disparity-based occlusion (resulting in amodal completion, [29]) may promote perceptual grouping through maximizing the efficacy of image-based grouping. Despite this clear distinction between low and higher stages of visual processing, our results do not show any trace of a higher-level form of image-based grouping. Instead, the durations of grouping during rivalry remain relatively stable under most conditions and appear mostly driven by eye-of-origin.

Although image-based grouping did not show any strong influences on dominance during rivalry in our experiments, we did find several subtle indications of influences of image content on grouping during rivalry. First of all, grouped face-parts were perceived for a larger portion of time than grouped gratings. Yet, dominance durations did not differ between grouped gratings and faces, suggesting that a percept based on grouped faces occurred more often, but did not last, on average, longer than a percept of grouped gratings. Interestingly, this bias towards grouped face percepts occurred only for face parts that were presented to the *same* eye. Thus, even when image-based grouping was present, it still appeared to be driven, or at least enabled, by eye-of-origin.

A similar dependency on early visual processing in the dominance of higher-level images has been demonstrated for the transitions during rivalry [31]. Arnold and his colleagues found that the spread of a transition during rivalry was slower when different facial regions were presented to different eyes. This suggests the involvement of monocular channels even for the dominance of higher-level images. A second consistent finding in our results is the modulation of grouping by the arrangement of stimuli across the visual field (as measured by overall occurrence as well as epoch durations). These results replicate our previous findings. For a full discussion on hemi-field effects on grouping during rivalry, see [17]. For now, it is important to note that the pattern in the hemi-field by eye-of-origin interaction is compatible with early visual processing.

When competing images were presented at a depth level that differed from the surround (Experiment 2), we found a small bias for perceiving mixed percepts containing upright face-parts compared to inverted face-parts. It is tempting to suggest that the presence of this face-inversion effect is due to the face-parts in Experiment 2 being presented along the vertical meridian, since no such effect of face-inversion was apparent in the first experiment. However, note that the difference in fraction dominance was very small (Fraction upright faces; average: 0.29, standard error: 0.02, Fraction inverted faces; average: 0.27, standard error: 0.02) and that we did not find any differences at the level of epoch durations for dominant faces. This shows that the effect of face inversion is not very robust. However, grouped faces were *not* dominant longer than grouped gratings for Experiment 2. Still, there was a trend of more overall grouping for faces relative to gratings. Yet, and again, this trend was only present during the eye-based grouping of faces. Therefore, we suggest an eye-level dependency for an effect that would previously have been attributed to higher-level processing [11].

Overall, our results show that the duration of and bias towards grouping during rivalry dominance is primarily determined by the eye-of-origin of the images. Still, image-content does undeniably play a role in grouping during rivalry, as is apparent from our own results [17] as well as the results of others (e.g. [10]–[12]). It is important to note, however, that results showing interocular grouping (simultaneous dominance of matched images presented to different eyes) have been used previously to state that dominance *cannot* be explained on a level of ocular dominance columns [11]. Nevertheless, the rivalling images, as well as the distance between them, tend to be relatively small in above-mentioned studies. This is also holds for our own studies on grouping, including the current study. This allows for explanations based on low-level lateral connections or effects based on the extra-classical receptive field since the rivalling elements should be processed relative close to each other in retinotopic coordinates. For example, Tong and colleagues [32] have suggested a model of binocular rivalry that includes feedback from a pattern-level of processing to a monocular level of processing. Importantly, they have also included lateral connections to account for a low-level, monocular version of image-based grouping. This latter component is similar to what is suggested from our data (also see [17]).

On the other hand, grouping during rivalry may not be established by early visual processing directly. Instead, dominance during rivalry, including grouped percepts, may involve higher-level brain structures that may modulate activity in lower-level structures by means of feedback [31]–[32]. Note that, if feedback plays an important role in grouping, we would expect extended grouping durations for face stimuli since they are thought to be processed in higher-level brain structures. For gratings stimuli, which are processed extensively in the early visual cortex, lateral connection are assumed to play a large role [12]. However, we found no major differences between grouping face stimuli and grouping gratings. This suggests that the contribution of feedback on grouping is probably much smaller than the contribution from these lateral connections. Moreover, based on our observation that image-based grouping is mostly absent in our study, it may even be that feedback is absent, at least in the conditions tested here. Our results therefore imply that the differences in grouping durations for different combinations of images might even reflect grouping on the basis of their low-level features, such as orientations and spatial frequency content, rather than a higher-level modulation.

Our results imply that image-based grouping is intertwined with eye-based grouping effects. We believe this reflects an early, monocular, processing stage for grouping during rivalry. The most straightforward source for this involvement is the ocular dominance columns in the early visual cortex. This is not necessarily incompatible with image-based grouping with features such as orientation [12]–[13]. Neurons within ocular-dominance columns are not only tuned to eye-of-origin but also to orientation and to spatial frequency (among others). This means they code different image aspects as well as the source of the image [33]–[35]. These columns may thus have all the machinery necessary for both the eye- and image-based grouping effects reported here. Theoretically, this would place image- and eye-based grouping during rivalry dominance at the same level of processing.

In summary, we have presented a large set of results on grouping during rivalry dominance that can be combined into a straightforward conclusion; grouping during rivalry is primarily based on low-level, early visual features such as orientation. A persistent pattern in our results is the importance of the eye-of-origin aspect of grouping during rivalry. Although image content (i.e. its feature content) does appear to play a role in grouping, it exerts its influence on a low level of the visual hierarchy, where it is intertwined with eye-of-origin information.
